# Odontogenic Sinusitis Presenting With Infraorbital Numbness and Progressive Maxillary Sinus Atelectasis

**DOI:** 10.7759/cureus.63891

**Published:** 2024-07-05

**Authors:** Dominic E McKenna, Vinson Fernandes, Ekambar Reddy

**Affiliations:** 1 Otolaryngology - Head and Neck Surgery, Southern Health and Social Care Trust, Craigavon, GBR

**Keywords:** rare anatomical variant, oroantral fistula, silent sinus syndrome, maxillary sinus atelectasis, infraorbital canal, odontogenic sinusitis, odontogenic maxillary sinusitis

## Abstract

Odontogenic sinusitis is the most common cause of isolated maxillary sinusitis. Accurate diagnosis is important to ensure optimal treatment. We discuss the unique presentation of a 55-year-old man with odontogenic sinusitis and associated infraorbital nerve neuropathy. We document his later development of chronic maxillary atelectasis and discuss the possible underlying pathophysiology linking this with his infraorbital neuropathy.

## Introduction

Odontogenic sinusitis (ODS) is an important cause of bacterial maxillary sinusitis, referring to the spread of infection through the sinus floor from an underlying dental aetiology such as infection or dentoalveolar surgery. It is common, driving up to 40% of chronic maxillary sinusitis and accounting for about half of the unilateral maxillary sinus opacification identified on CT [1]. Recognition is important as its pathogenesis, treatment, and outcomes differ greatly from that of chronic rhinosinusitis.

Despite its prevalence, it is underrepresented in the literature [2]. A recent international consensus statement highlighted the importance of multidisciplinary collaboration between otorhinolaryngology and dental specialists in the diagnosis of ODS [1].

The infraorbital nerve (ION) is a branch of the maxillary nerve, arising from it in the inferior orbital fissure, passing through the infraorbital groove and canal to terminate at the infraorbital foramen in the face. It supplies ipsilateral sensation from the lower eyelid to the upper lip [3].

Iatrogenic damage to the ION is rare in routine sinus surgery, although it is at risk in more extended procedures required, for example, following trauma or in the treatment of malignancy or inverted papilloma. Previously thought to be rare, anomalous protrusion of the infraorbital canal (IOC) into the maxillary sinus has been the focus of a number of recent cross-sectional imaging studies, demonstrating an incidence between 8.8% and 15.7% [4].

The functional importance of the ION is underpinned by its detrimental impact on the quality of life when damaged through trauma [5].

We discuss a unique case of a 55-year-old man with an anomalous IOC who developed ION neuropathy from ODS. He later demonstrated evidence of chronic maxillary atelectasis (CMA). We also discuss the important clinical aspects of this case, as well as the possible unifying underlying pathophysiology behind these rare entities.

## Case presentation

A 55-year-old man presented to the emergency department with an eight-week history of progressive right-sided facial pain and numbness, extending from his right lower eyelid to his upper lip. He had mild associated facial swelling, nasal obstruction, and purulent nasal discharge. He worked in a factory with frequent exposure to carbonaceous soot. He denied any history of trauma. He has no medical background and was a 30-pack-year smoker. He was systematically well, with mildly increased inflammatory markers (C-reactive protein of 37 mg/L (0-5 mg/L) and white cell count of 14.3x10^9^/L (4-10x10^9^/L).

**Figure 1:**
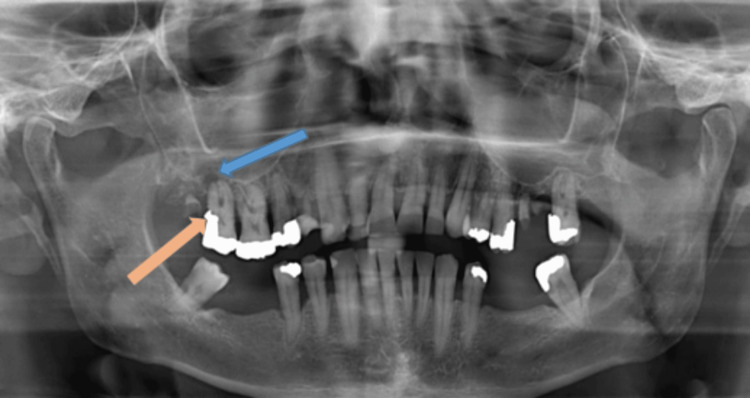
Orthopantomogram: Periapical lucency right upper second molar (blue arrow) with caries in the crown (orange arrow)

An orthopantomogram (OPG) showed a small periapical lucency around his maxillary second molar tooth, consistent with a dental abscess (Figure 1). He was commenced on oral co-amoxiclav, attended his community dentist, and had the removal of both right maxillary premolars along with his first and second maxillary molars. Following dental extractions, there was no evidence of dental suppuration; however, his facial and rhinological symptoms persisted.

**Figure 2:**
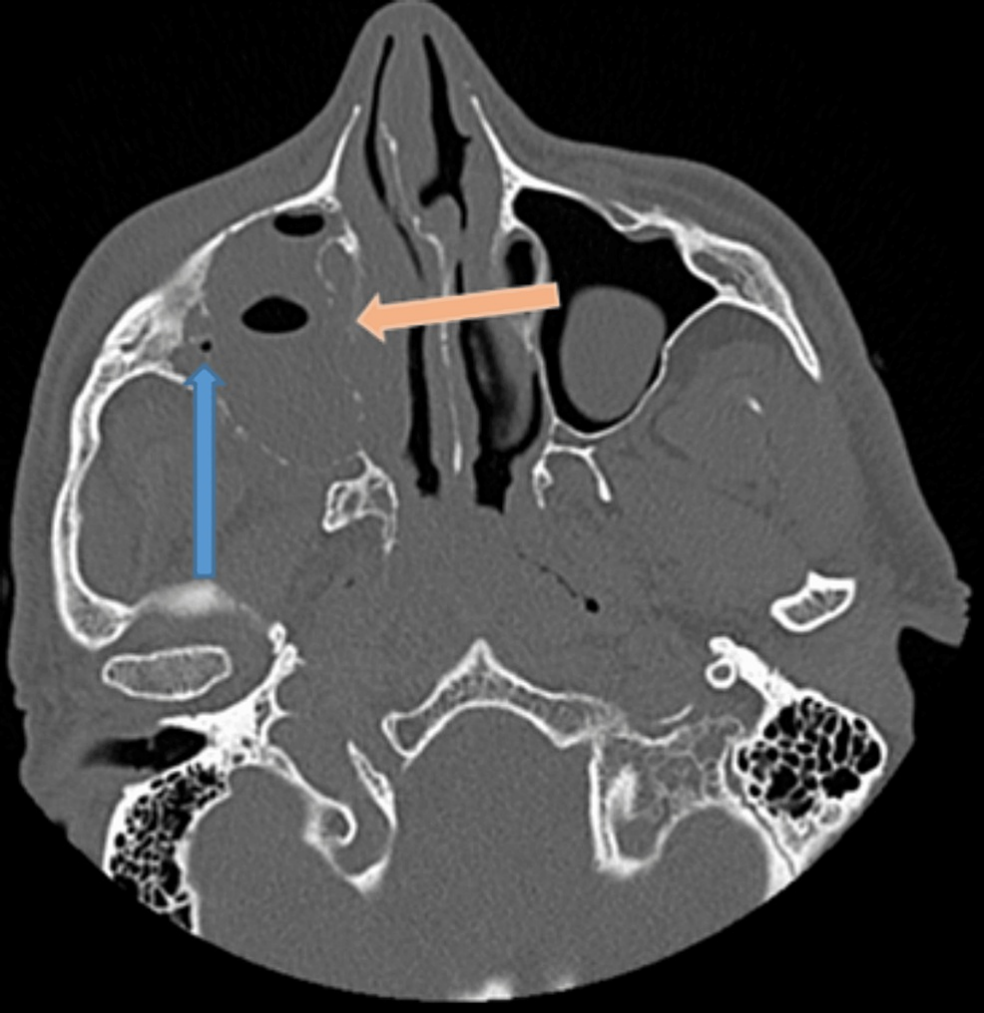
CT sinus – axial image Sub-total opacification of the right maxillary sinus with thinning and dehiscence of the medial and lateral sinus walls (orange arrow). There is a suggestion of an intra-sinus IOC with bony dehiscence (blue arrow).

**Figure 3:**
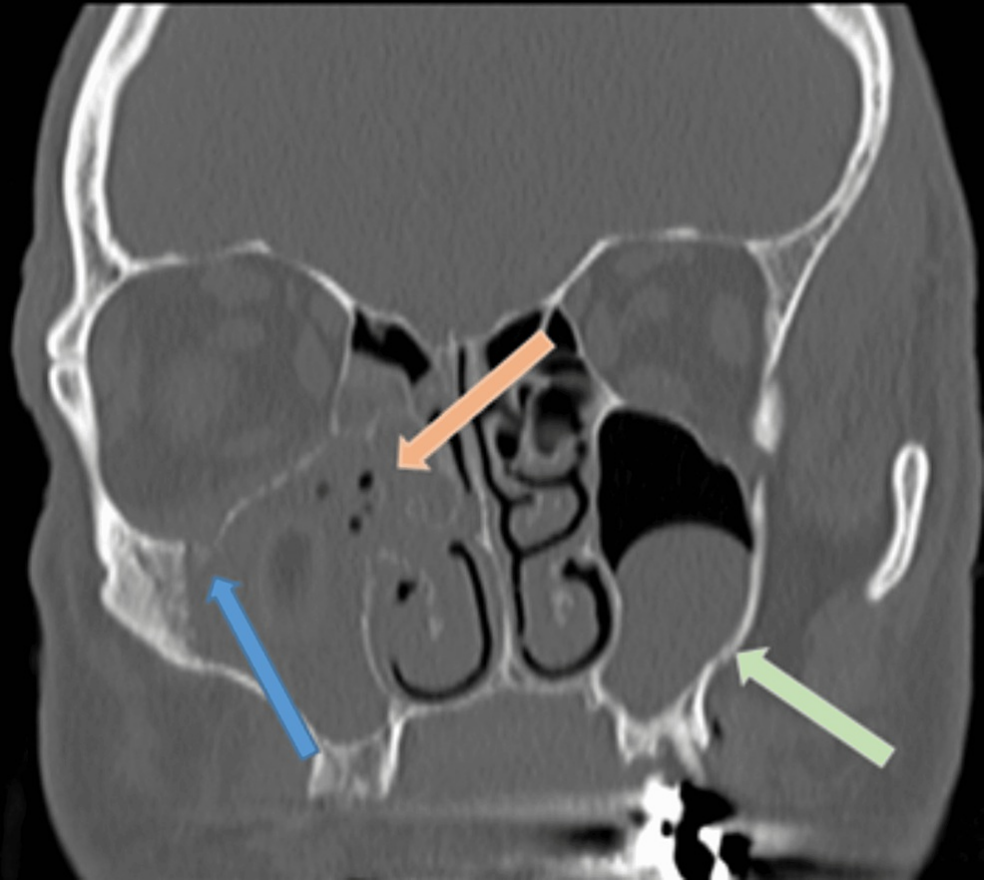
CT sinus – coronal image Adjacent subtotal osteomeatal unit opacification with anterior ethmoid and frontal sinus opacification (orange arrow) thinning and dehiscence of the medial sinus wall and orbital floor are visible. Again, a laterally positioned intra-sinus IOC is suggested (blue arrow). The well-defined polypoid mass in the left maxillary sinus is likely an incidental mucus retention cyst (green arrow).

He re-attended the emergency department four weeks later. Following a discussion with the maxillofacial surgeons, he had computed tomography (CT) of his paranasal sinuses that showed almost complete opacification of his right maxillary sinus, frontal sinus, and ipsilateral osteomeatal unit (Figure 2). There was also evidence of thinning and dehiscence of the medial and lateral sinus walls, as well as his orbital floor. Although interpretation is difficult owing to the adjacent opacification, there was a suggestion of a dehiscent intra-sinus IOC (Figure 3).

On review by ENT, rigid nasal endoscopy revealed a generally congested and pus-filled right middle meatus. He had ongoing facial numbness over the distribution of his ION. He was apyrexic with no orbital symptoms or facial asymmetry. He was commenced on topical saline irrigation, topical corticosteroid drops, and a further course of antibiotics (co-amoxiclav and metronidazole) to good clinical effect. There was resolution of his facial pain and nasal discharge; however, his facial numbness continued.

**Figure 4:**
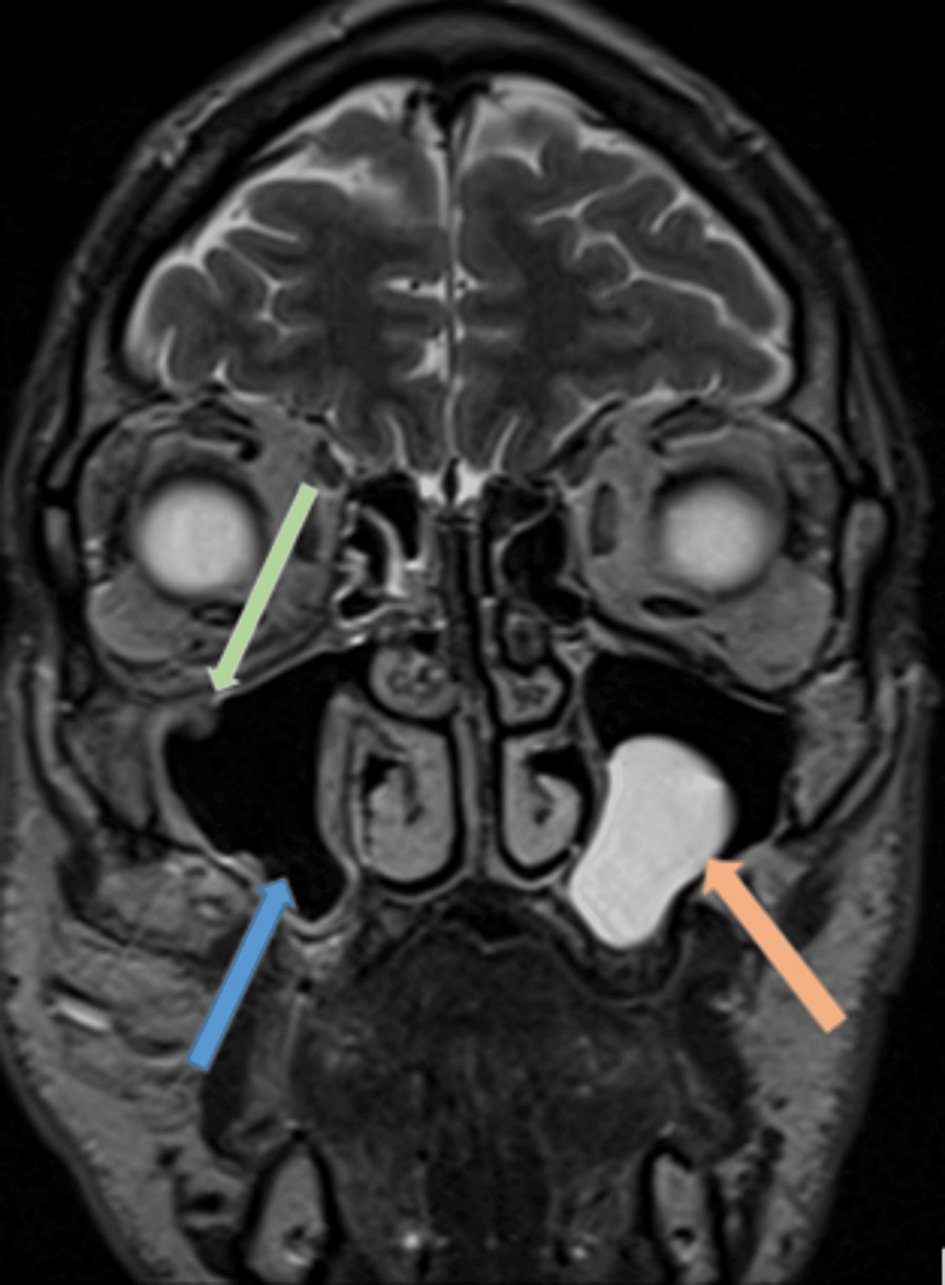
T2-weighted MRI – coronal image Significant reduction in right maxillary sinus opacification (blue arrow). The ION is visible within the maxillary sinus (green arrow). Stable polypoidal mucosal thickening in the left maxillary antrum (orange arrow).

**Figure 5:**
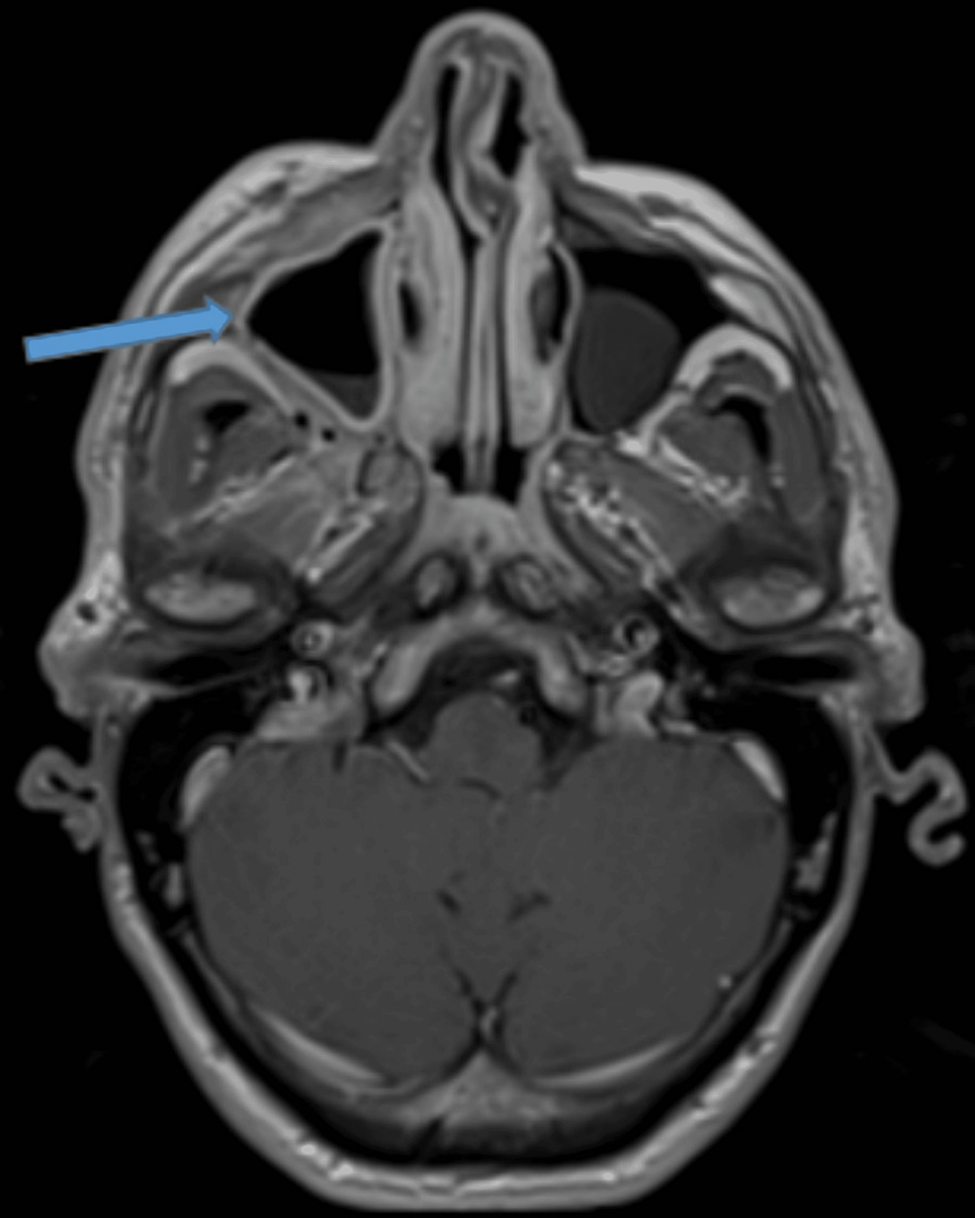
T1-weighted post-contrast MRI – axial image Enhancing inflammatory change within the soft tissues of the right maxillary wall (blue arrow).

Red flag outpatient magnetic resonance imaging (MRI) showed improvement in his underlying right maxillary sinus opacification, with ongoing residual enhancing inflammatory change around the maxillary sinus walls. It also confirmed the presence of a prolapsed ION within the maxillary sinus (Figures 4, 5). Given his symptomatic improvement, he was discharged from ENT with a diagnosis of resolving odontogenic sinusitis.

Six months later, he was re-referred to otorhinolaryngology from primary care with ongoing numbness and neuropathic pain in the distribution of the ION requiring amitriptyline. He had no associated nasal, dental, or orbital symptoms, and his nasal examination was unremarkable.

**Figure 6:**
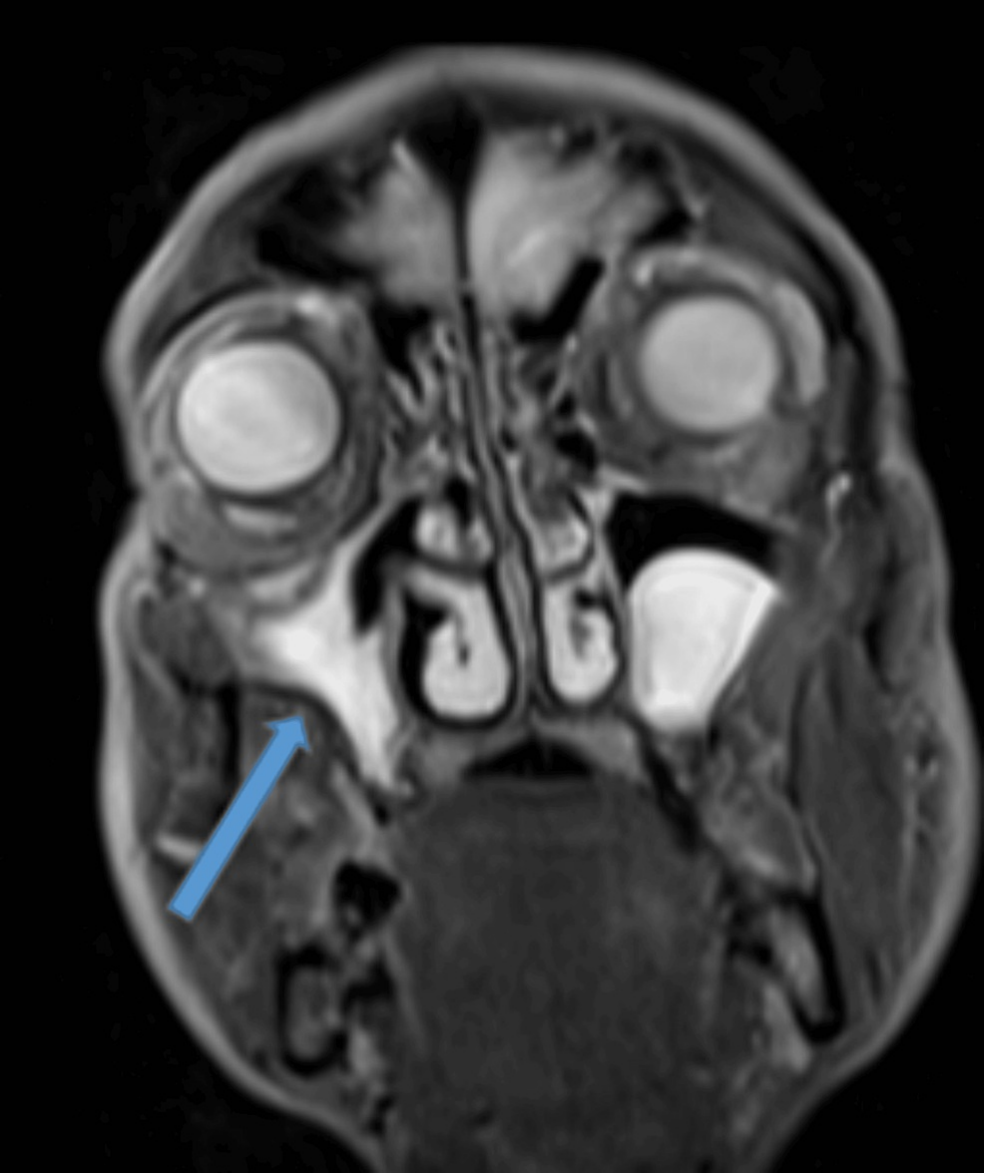
T2-weighted turbo inversion recovery magnitude (TIRM) MRI – coronal image Subtotal opacification of the right maxillary with loss of the sinus cavity volume and in the drawing of the lateral sinus wall (blue arrow).

**Figure 7:**
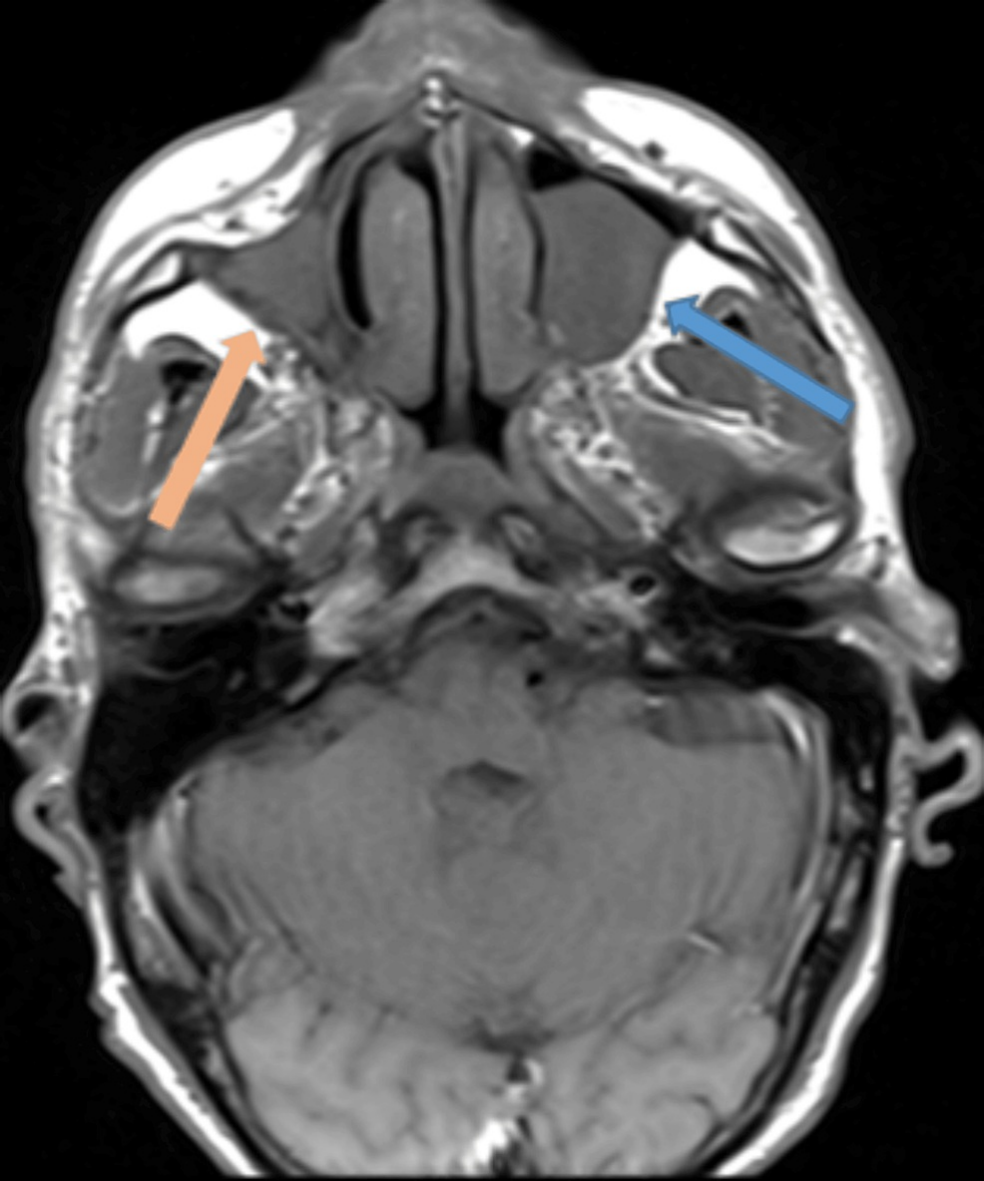
T1-weighted turbo spin echo (TSE) MRI – axial image Near complete opacification of the right maxillary with loss of the sinus cavity volume (orange arrow). There has been mild progression in the left maxillary sinus polypoidal thickening (blue arrow).

A repeat MRI scan demonstrated complete opacification of his right maxillary sinus with evidence of sinus wall atelectasis (Figures 6, 7). Urgent right-sided functional endoscopic sinus surgery was organised.

**Figure 8:**
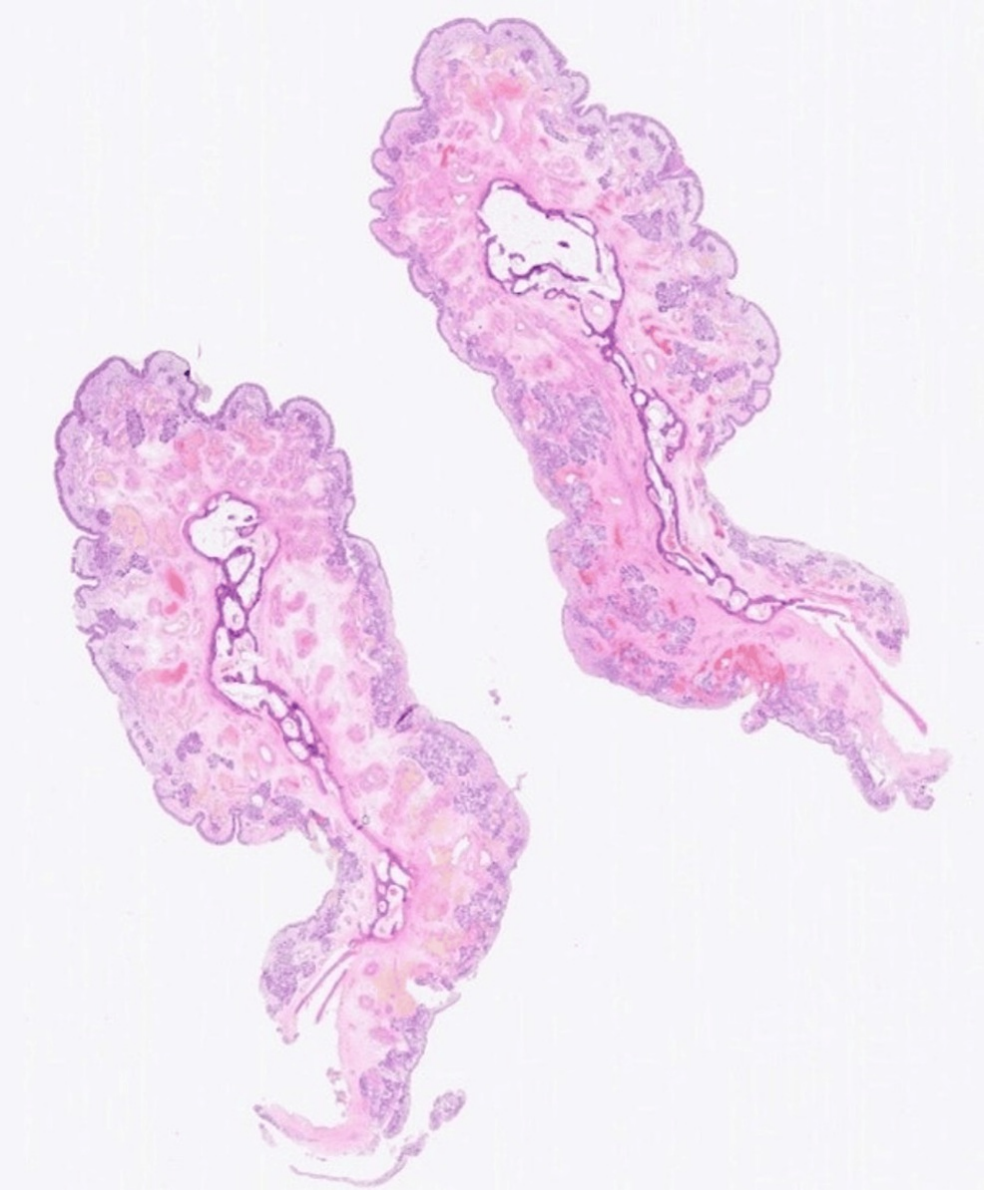
Normal inferior turbinate bone histology

**Figure 9:**
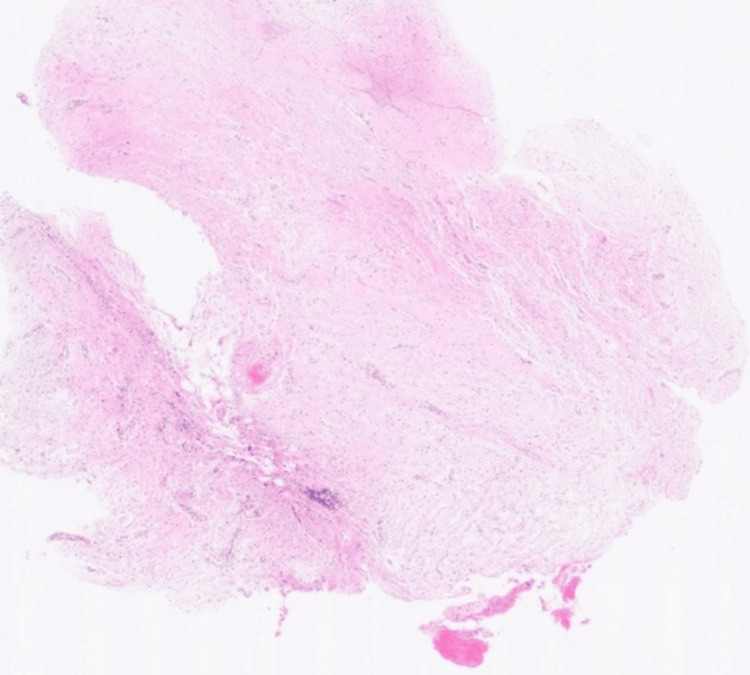
Hypertrophic maxillary sinus mucosa with no evidence of neoplasia

The procedure was challenging owing to his small contracted maxillary sinus. Unusually thick bone around the maxillary sinus opening was present, requiring the removal of the posterior two-thirds of his inferior turbinate to allow for a posterior antrostomy. Histological examination of his resected inferior turbinate, along with hypertrophic mucosa in the maxillary sinus, showed no evidence of neoplasia or granulomatous inflammation (Figures 8, 9).

Following his surgery, there was a significant improvement in his facial symptoms, with resolution of his neuropathic pain allowing discontinuation of amitriptyline. Nine months following surgery, he has ongoing right infraorbital numbness without associated facial asymmetry and nasal or orbital symptoms.

## Discussion

To our knowledge, our case represents the first documented instance of both ODS-related infraorbital neuropathy and ODS-related maxillary sinus atelectasis.

ODS is a common cause of maxillary sinus opacification. It usually arises from the iatrogenic or infectious violation of the Scheiderian membrane (Table 1). This is a bilaminar membrane unique to the maxillary sinus, lined with ciliated columnar epithelium internally and periosteum externally [6].

**Table 1:** Odontogenic sinusitis – aetiological factors [7]

Most common causes	65.7%	Iatrogenic - impacted tooth, artificial implants, dental amalgams, oroantral fistula
	16%	Apical periodontitis
	8.3%	Marginal periodontitis
	5%	Apical granulomas
	2.5%	Odontogenic cysts
Most frequently affected maxillary teeth	35.6%	First molar
	22%	Second molar
	17.4%	Third molar
	14.4%	Second premolar

The diagnosis of ODS requires confirmation of the presence of sinusitis, in addition to identification of a likely odontogenic source of this infection [1].

ODS shares symptomatology with chronic rhinosinusitis with anterior/posterior nasal discharge, hyposmia, nasal obstruction, and facial pressure common. Although foul smell is more specific for ODS, it is not pathognomonic, and some patients will have asymptomatic disease. Nasal endoscopy is the most important investigative tool. It can help confirm an infectious aetiology of any sinus opacification demonstrated on CT imaging [1]. ODS should always be suspected in unilateral maxillary sinus disease although there are other important differentials that should always be considered (Table 2).

**Table 2:** Most common causes of unilateral sinus opacification [8]

Most common causes	45%	ODS
	36%	Non-odontogenic inflammation - chronic rhinosinusitis with or without polyps, antrochoanal polyp, allergic fungal rhinosinusitis, mycetoma
	17%	Inverted papilloma
	2%	Malignancy

An odontogenic source is confirmed by the identification of an oroantral fistula or by making the diagnosis of endodontic disease, through imaging or clinical pulp testing [1]. A multidisciplinary approach is vital to allow concurrent treatment of both the sinusitis and the underlying dental source. This usually entails oral antibiotics and dental surgery in combination with endoscopic sinus surgery [9].

Chronic maxillary atelectasis (CMA) is a rare and underdiagnosed condition characterised by a progressive reduction in maxillary sinus volume, with inward bowing of its membranous or bony walls [10]. Pathologically, the most commonly accepted theory describes chronic hypoventilation of the sinus from mucus drainage obstruction, leading to negative pressure and subsequent thinning and retraction of the sinus walls. This is akin to the pathological process in the ear, where chronic Eustachian tube dysfunction leads to negative middle ear pressure and subsequent retraction of the tympanic membrane [11,12].

CMA has been divided into three stages depending on the anatomical and clinical features (Table 3).

**Table 3:** Stages of chronic maxillary atelectasis [13]

Stage of CMA	Pathological features
I	Isolated membranous deformity with laterisation of the posterior maxillary fontanelle
II	Bony deformity with inward bowing of the maxillary sinus walls
III	Clinical deformity evident with change in orbit position +/- midfacial deformity

Symptoms of CMA reflect the underlying rhinological disease, with facial pain and pressure, anterior and posterior nasal discharge, congestion, and headache all described. In stage III disease, loss of orbital floor integrity leads to enophthalmos and facial deformity.

Silent sinus syndrome (SSS) is a similar condition, with some authors stating that it lies on the same clinical spectrum as CMA [11]. Classic SSS differs from stage III CMA by the absence of rhinological symptoms although this nomenclature is not strictly followed in the literature.

CMA stage I and II are usually treated with an endoscopic middle meatal antrostomy, aiming to restore antral drainage and ventilation. Care must be taken as loss of maxillary sinus volume and depression of the orbital floor may increase the risk of orbital entry. CMA stage III or SSS may require additional orbital reconstruction in the presence of diplopia or cosmetic concerns that do not resolve with sinus surgery alone [14].

The constellation of unique anatomical and pathological findings in our case may help shed light on the underlying pathogenesis of CMA. We would propose that the persistent negative pressure induced by the ODS-driven osteomeatal unit dysfunction led to the resorption of the bony IOC within the maxillary sinus cavity, exposing the IOC to infection-related neuropathy. This same pathological process also led to bony resorption of the orbital floor, as well as the medial and lateral sinus walls. The subsequent drawing of the bony maxillary walls and loss of sinus volume is best described as stage II CMA.

Of note, upper alveolar nerve numbness has been reported in association with SSS, possibly related to associated bony sclerosis causing nerve compression [15]. We feel that a similar process is unlikely in our case as this patient's ION neuropathy preceded radiological evidence of CMA and would be better explained as infectious-related neuropathy.

## Conclusions

This case represents a novel description of ODS causing ION neuropathy in the presence of an anomalous IOC. It serves to highlight the importance of considering ODS as a cause of atraumatic IOC neuropathy. In addition, this case is unique in that it also documents the development of CMA in the presence of ODS, contributing to the understanding of this uncommon entity.
